# Apple Peel Deformity and Malrotation of Gut: Autopsy Findings of a Rare Cause of Mortality in Utero

**DOI:** 10.5146/tjpath.2021.01533

**Published:** 2022-01-21

**Authors:** Deepti Mutreja, Sharanjit Singh

**Affiliations:** Department of Pathology, Armed Forces Medical College, Pune, India

**Keywords:** Intestinal Atresia, Apple peel deformity, Autopsy

## Abstract

One-third of all intestinal obstructions in the newborn are caused by atresias. The most common site is the duodenum followed by jejunoileal and colonic locations. Herein we report the autopsy findings of a rare case of jejunoileal atresia associated with malrotation of gut. Autopsy performed on a 36 weeks old male fetus still birth, born of a non-consanguineous marriage, demonstrated jejunoileal atresia with apple peel deformity and malrotation of gut. Although the diagnosis was established in the prenatal period, in utero fetal demise occurred before definitive surgical intervention could be done. This case highlights the importance of early diagnosis and intervention.

## INTRODUCTION

Intestinal atresia is a rare congenital anomaly, the incidence of which ranges from 1.3 to 3.5 per 10,000 live births (1,2). Twenty percent of atresias have accompanying chromosomal anomalies. The commonest site is the duodenum, accounting for half the cases reported (2). Jejunoileal atresia is extremely rare and accounts for only 3 per 10000 live births (3). Apple peel syndrome refers to the ileal segments of small intestine wrapped around an atretic jejunal segment in a spiral manner resembling an apple peel (4). Also known as “pigtail-like syndrome” it is extremely rare and reported to occur in less than 5% of cases. Apple peel atresia may occur in isolation; however, it has been associated with malrotation of gut, and other anomalies (5). We present autopsy findings of a rare case of apple peel deformity due to jejunoileal atresia associated with malrotation of gut. The precise aetiology of intestinal atresias is still unidentified and various theories propose that these result either from vascular compromise to the gut later in gestational life or due to loss of recanalization in early intrauterine life (6). With the report of this case, we bring focus on this uncommon condition. A thorough review of the literature reveals that very few cases of such association have been reported in literature.

## CASE REPORT

**Figure 1:**
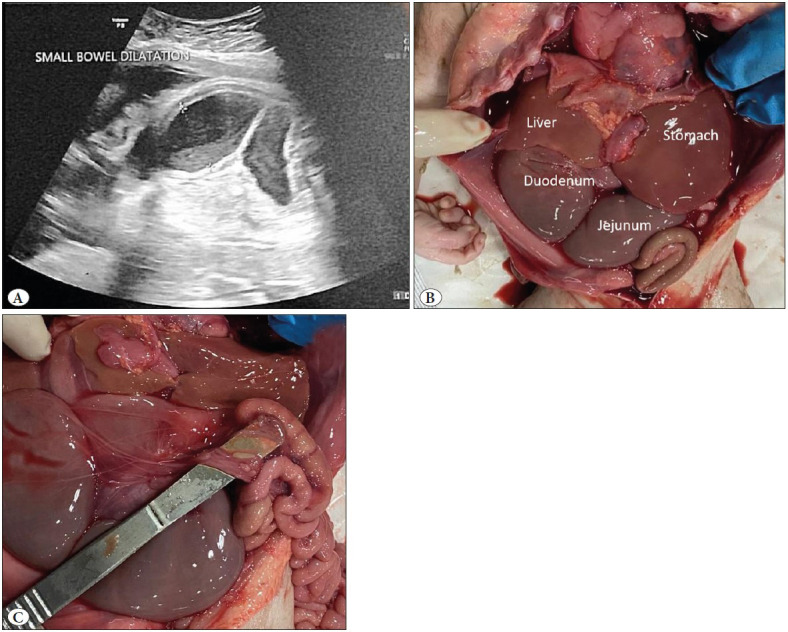
**A)** Ultrasound scan showing dilated gastric fundus bubble with dilated proximal bowel. **B)** Dilated and congested stomach, duodenum and jejunum. **C)** Scalpel head showing atretic intestinal segment.

A 2.22 kg male stillborn neonate was delivered vaginally following induction of labour to 31-year-old gravid 3, para 2 mother at 35 weeks 5 days period of gestation. The parents were in a non-consanguineous wedlock. The mother was a known case of hypothyroidism on eltroxin. There was previous history of still birth during first pregnancy. Dual and triple screen tests were negative. Maternal antenatal routine investigations were essentially normal. Antenatal ultrasonography in the first and second trimesters was normal. However, during a routine visit in the third trimester, the ultrasound scan showed a dilated gastric fundus bubble, and dilated proximal bowel with a maximum diameter of 2.5 cm (Figure 1A). No associated anomalies were seen. Paediatric surgery review was sought and surgery was planned in the immediate post-natal period. Repeat ultrasound after one week confirmed jejunal atresia with intra-amniotic hematoma which was fetal in origin with severe fetal bradycardia. Maternal blood investigations revealed normal haematological parameters but liver enzymes were elevated (AST was 412IU/L and ALT was 568 IU/L). The patient was taken up for immediate delivery but repeat ultrasound in the labor room showed no fetal activity. A neonatal autopsy was carried out.

**Figure 2:**
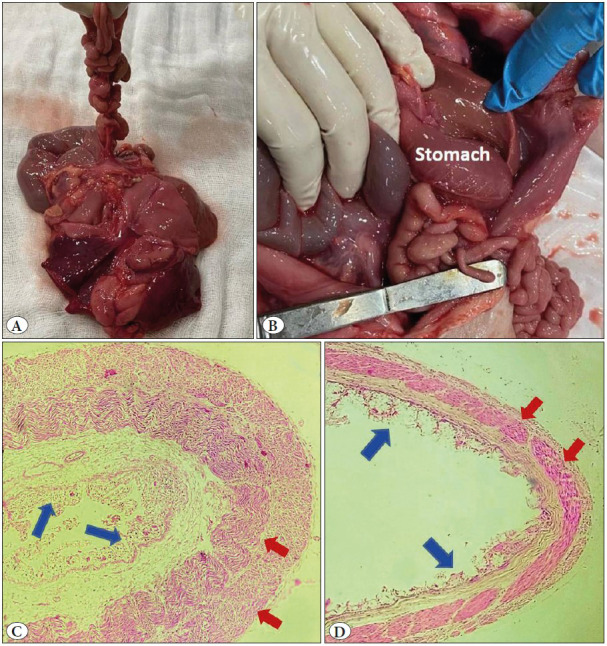
**A)** Distal small intestine coiled around a fibrous band (apple peel deformity). **B)** Malrotation of gut with appendix lying on left side. **C)** Section from dilated duodenum proximal to site of atresia showing partially autolysed mucosa (blue arrows) with thickened muscle layers (red arrows) (H&E; x100). **D)** Section from distal small bowel behind the atretic site showing partially autolysed mucosa (blue arrows) along with normal thickness of muscle layers (red arrows) (H&E; x100).

Pertinent findings on autopsy included a macerated male fetus whose anthropometrical measurements were normal for age and sex. Development of facial features, thorax, and extremities appeared appropriate for the gestational age. No anatomic defects were identified on gross evaluation. Head, chest and abdominal circumference were normal for 35 weeks gestational age. Cephalhaematoma with overriding of skull bones was noted. On opening the body, the most remarkable finding was marked distension, dilatation and congestion of the duodenum and jejunum that ended in a blind pouch (Figure 1B,C). The distal small intestine was shortened and coiled around a fibrous band (apple peel deformity) (Figure 2A). There was malrotation of gut with appendix lying on the left side (Figure 2B). The distal small intestinal segment although shortened was patent and contained meconium. Gross examination of other organs including the heart, lungs, kidneys was unremarkable.

Histopathological examination of most of the organs showed autolytic changes Sections from the dilated duodenum showed autolytic mucosa with a markedly thickened muscle layer (Figure 2C). Sections from the distal small bowel showed partial autolytic changes in the mucosa with normal thickness of the muscle layer (Figure 2D). Sections from the atretic segment showed fibromuscular tissue only. Examination of placental tissue was unremarkable. Umbilical cord showed normal three-vessel morphology without any evidence of funisitis.

Fetal tissue submitted for cytogenetic analysis showed normal cytogenetics.

It is certified that ethical approval of ethics committee of Command Hospital Air Force Bangalore and consent of next of kin of deceased was sought prior to publication of this study.

## DISCUSSION

One-third of all intestinal obstructions in the newborn are caused by atresias (1,2). The autopsy in this case demonstrated jejunoileal atresia with apple peel deformity and malrotation of gut. The etiology of atresia has been postulated by various authors. Duodenal atresia is believed to result from failure of canalization of gut lumen and generally occurs early in pregnancy within the sixth to the eighth week of gestation whereas jejunoileal atresia is postulated to occur in the second or third trimester as a result of vascular injury to already canalized gut (6). Jejunoileal atresias generally are not associated with other congenital anomalies whereas duodenal atresia is common in Down’s syndrome (7). Maternal use of antimigraine drugs namely pseudoephedrine alone or in combination with acetaminophen, ergotamine and caffeine have been linked to increased risk for development of small intestinal atresia (8). There was no history of such drug intake in our case.

Jejunoileal atresia could be single (>90%) or multiple and may be familial (9). There was a history of fetal demise in a previous pregnancy in this case; however, it was not investigated. There may be associated intrauterine growth restriction, gastroschisis, volvulus, malrotation of the gut, and cystic fibrosis (10). Our case had malrotation of the gut as an associated anomaly. Jejunoileal atresia is traditionally classified as per Grosfeld subtypes. Type I is mucosal in which the lumen is blocked by an intact membrane. Type II has atretic bowel ends which are connected by a fibrous cord. Type III has two variants. Type IIIA has an obvious gap between disconnected segments. Type IIIB is the rarest form with apple peel deformity while Type IV has multiple obstructions resulting in sausages on string appearance and is the least common variant (11). This neonate also had type IIIB deformity. Almost one-third of JIA and duodenal atresia cases can be identified during the prenatal period with ultrasonography (12). In our case, maternal ultrasound up to the 29th week was reported as normal but routine third trimester ultrasound showed dilated bowel loops. Dilated bowel loops are highly indicative of bowel obstruction due to a congenital disorder, intestinal malrotation, volvulus, and meconium plug syndrome (13).

The most commonly accepted theory for pathogenesis of intestinal atresia is based upon work by Louw and Barnard (6,14). They demonstrated and reproduced all models of atresia after ligation of mesenteric vessels at various levels in canine models, thus supporting their theory of vascular insufficiency. Our case had malrotation of gut that possibly led to vascular insufficiency leading to jejunoileal atresia.

What might have caused intrauterine fetal demise in the current case? It is postulated following obstruction, the raised bile acids in fetal bloodstream, may have triggered a fetal arrhythmia, or marked dilatation of the stomach, duodenum and proximal jejunum in this case may have triggered bradycardia and ultimately sudden death resulting from high vagal tone (15,16).

With respect to prenatal diagnosis, repeated ultrasound imaging is useful to assess the progress and development of atresia in the foetus. If undetected, the neonate may present with signs and symptoms of high bowel obstruction several hours after delivery. Despite the high mortality, it can be treated and managed with appropriate early surgical intervention (17).

Our autopsy case report presents a rare and complicated variant of jejunoileal atresia associated with malrotation of gut. This case highlights the importance of early diagnosis and intervention.

## Conflict of Interest

The authors declare no conflict of interest.

## Funding

None
